# Endometriosis Mimicking a Cecum Mass with Complete Bowel Obstruction: An Infrequent Cause of Acute Abdomen

**DOI:** 10.1155/2019/7024172

**Published:** 2019-01-31

**Authors:** Gabriel A. Molina, Darwin R. Ramos, Alberto Yu, Patricio A. Paute, Paul S. Llerena, S. Alexandra Valencia, Jose V. Fonseca, Jhonatan F. Morillo, Sandra C. López, Bernardo M. Gutierrez

**Affiliations:** ^1^PGY4 Resident General Surgery, P.U.C.E, Quito, Ecuador; ^2^PGY1 Resident General Surgery, U.C.E, Quito, Ecuador; ^3^PGY3 Resident General Surgery, P.U.C.E, Quito, Ecuador; ^4^USFQ, School of Biological and Enviromental Sciences, Quito, Ecuador

## Abstract

Endometriosis is a common entity among fertile women which unfortunately manifests through variable symptomatology. Intestinal involvement in endometriosis is quite common and can simulate several diseases such as Crohn's disease, appendicitis, tubo-ovarian abscess, or malignant tumors. Intestinal obstruction due to endometriosis is rare, and preoperative diagnosis is difficult because the signs and symptoms are nonspecific and can be easily confused. In the case of patients without a history of endometriosis, diagnosis is further complicated. We present a case of a 41-year-old female patient. She presented to the emergency room with complete bowel obstruction and a mass in the cecum. Surgery was decided, and the patient underwent full recovery. Endometriosis was the final diagnosis for the observed condition.

## 1. Introduction

Endometriosis is the presence of benign endometrial tissue in extrauterine sites, a condition with a prevalence of up to 10% to 15% of the general population of women [1, 2]. However, complications in its diagnosis such as the need for visualization to confirm its occurrence suggest that the real prevalence could be higher [1]. It usually manifests within the pelvis, but it has been observed on other organs [2]. One of such nongynecological cases, known as intestinal endometriosis, manifests in 3% to 37% of endometriosis patients and commonly involves the rectum and sigmoid colon [3]. Endometriosis of the gastrointestinal tract is usually asymptomatic, but symptoms such as abdominal pain, distention, vomiting, diarrhea, constipation, dyspareunia, and hematochezia could occur in some cases [3]. These symptoms can mimic other pathologies like Crohn's disease, appendicitis, tubo-ovarian abscesses, intestinal obstructions, or malignancies [3], especially in patients without a previous history of endometriosis [3, 4].

Endometriosis is common yet complex, as it is associated with a broad spectrum of clinical presentations. Despite chronic pelvic pain being common, women having endometriosis in unusual sites or experiencing complications may present with acute abdominal pain in up to 8% of the cases and require urgent medical attention [1, 2].

## 2. Case Report

**Figure 1:**
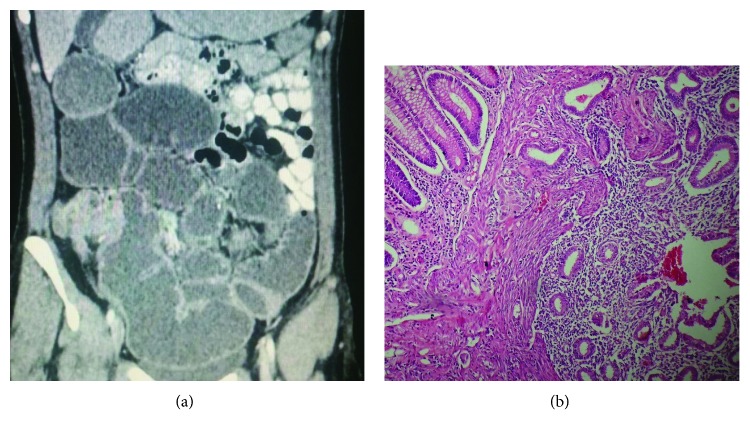
(a) Tomography revealing a cecum mass with complete bowel obstruction. (b) Colonic epithelium, adjacent to endometrial glands and stromal tissue.

**Figure 2:**
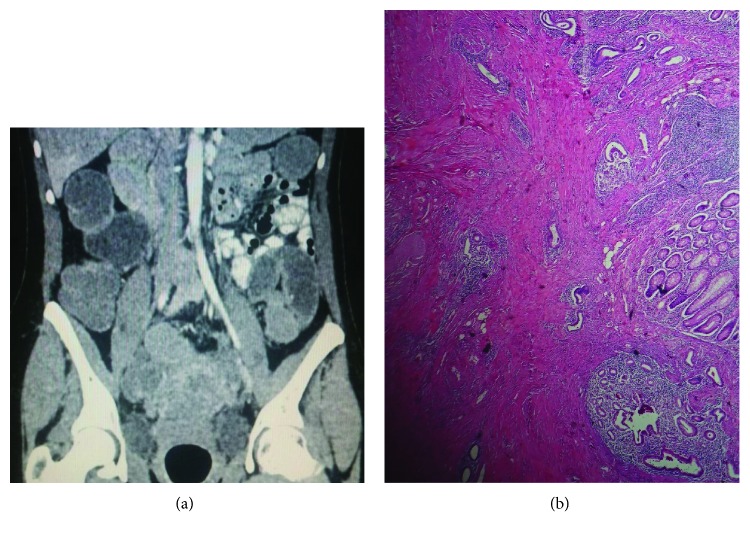
(a) Right adnexal mass surrounded by loops of the small bowel. (b) Endometrial tissue in the muscular layer of the cecum.

**Figure 3:**
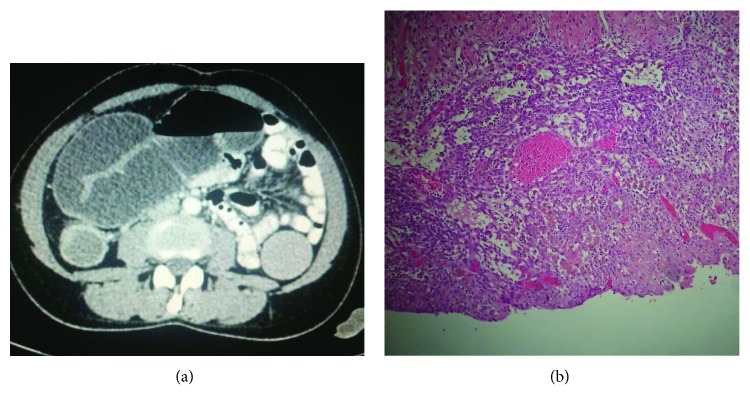
(a) Dilated loops of the bowel with complete intestinal obstruction. (b) Ovarian tissue with implants of endometrial glands, surrounded by siderophages.

The patient was a 41-year-old female with past medical history of appendectomy and dysmenorrhea. She presented to the emergency department with nausea, severe vomiting, and acute pain in the lower abdomen. She reported having experienced asthenia and weight loss for one month. On clinical examination, abdominal distension and tenderness were discovered. Blood tests revealed leukocytosis with neutrophilia, and a contrast-enhanced abdominal computed tomography (CT) showed a 7 × 7 × 4 cm hyperenhanced mass in the cecum that caused complete bowel obstruction (Figure 1(a)). Also, a 5 × 3 × 3 cm right adnexal mass that compromised the ovary with intimate contact with the uterus was found (Figure 2(a)). Furthermore, the CT showed dilated loops in the small bowel (>4 cm), some of which had an enlarged wall thickness and presence of intraluminal fluid stasis (Figure 3(a)).

With these findings, particularly the observation of a mass through the CT scan, and due to the evident weight loss that the patient had undergone, neoplasia could not be ruled out. Surgery was decided, and at laparotomy, a volume of 200 ml of inflammatory fluid was found in the cavity. Most of the loops of the distal ileum were dilated, and a 7 × 7 × 3 cm cecum mass was discovered, which compromised the ileocecal valve and caused complete bowel obstruction. Surgical decision was straightforward, the cecum mass was completely resected, and a right hemicolectomy was executed. An ileocolic anastomosis was also performed during the procedure. Furthermore, the right adnexal mass that was previously identified through the CT scan (measuring 4 × 3 × 2 cm) was observed to be firmly attached to the ovary and the fimbriae and displayed a pale external capsule surrounded by a cystic component. Gynecology consultation was required, and due to the size of the mass and its characteristics, surgical removal of the right adnexal mass was performed. After completion, closure of the abdominal wall was performed, and the remainder of the procedure continued without any complications.

Pathology revealed a 4 × 3 × 2.5 cm blueish heterogenic mass that occluded 90% of the lumen of the cecum and the ileocecal valve. Microscopy revealed that the colon wall was invaded by glands and endometrial stroma. The colonic epithelium showed inflammatory changes and was negative for malignancy (Figures 2(a) and 2(b)). In the ovarian parenchyma, an endometrial cyst was discovered, covered with siderophages. Glands and endometrial stroma were observed in the fallopian tube as well (Figure 2(c)).

The postoperative course of the patient was uneventful. She initiated clear liquids a day after surgery and was discharged once full diet was resumed. On follow-up controls, the patient was completely asymptomatic, without any pain or complications.

## 3. Discussion

Ever since Sampson et al. theorized in 1927 that endometriosis could result from retrograde deposition of endometrial remains during menstruation, various theories for the cause of this condition have been proposed, including the coelomic metaplasia of the peritoneum or the dissemination of endometrial particles through lymphatic and hematogenous pathways [2]. However, the true pathogenesis of endometriosis remains unknown [5]. The most common sites of endometriosis are the ovaries, cul-de-sac, and uterosacral ligaments [1, 2], while atypical nongynecological sites for the disease include the gastrointestinal, appendiceal, urinary tract and abdominal wall tissues, with additional reports on the pulmonary tract, lymphatic system, skin, musculoskeletal system, and central nervous system [3]. These atypical sites pose a particular challenge for accurate diagnosis [5].

Endometriosis is present in up to 12% of fertile women, with the number of cases diagnosed peaking between the ages of 29 and 39 years [2]. It can present with dysmenorrhea, dyspareunia, deep pelvic pain, infertility, or lower abdominal pain [2]. These symptoms appear more commonly in women of reproductive age [3] and may depend on the location and depth of the disease; however, the extent of the disease may not necessarily be correlated with the severity of the symptoms [3, 4]. Women may, in rare occasions, suffer from acute abdominal or pelvic pain severe enough to cause them to seek emergency medical care [6], as reported in our case. Surgical procedures associated with the condition are not uncommon. Golditch et al. described 340 patients with endometriosis over a 10-year period from 1955 to 1964 and reported that 8% were presented as having acute surgical emergencies [1].

The involvement of the bowel in intestinal endometriosis is typically associated with the disease at other sites [7], as presented by our patient. It is noteworthy that the cecum is involved in less than 5% of all intestinal endometriosis cases [7], as the more common sites include the rectosigmoid, followed by the proximal colon, small intestine, and appendix [3] according to the order of frequency of occurrence.

Bowel obstructions due to endometriosis are rare, occurring in less than 1% of all patients [2]. When this occurs, urgent treatment is suggested [8]. However, in the case of patients without a prior history of endometriosis, the differential diagnostic procedures can cover a broad spectrum, and making the correct clinical and radiologic diagnosis in an emergency setting can be challenging. Due to the unspecific symptoms of endometriosis and the probability of misidentification of observed masses in CT scans [9], the diagnosis is only made after surgical and histopathological analysis [10, 11], as it occurred with our patient. Prompt and accurate clinical and radiological evaluation is necessary because complications of endometriosis, such as bowel obstruction or perforation, may require immediate surgical intervention [3, 4].

In our case, it was not possible to establish an accurate preoperative diagnosis due to the clinical features and the images seen in the CT. However, due to the intestinal obstruction, surgery was decided and the patient successfully recovered. Our case also demonstrates in a unique way that despite its rarity, surgeons should be aware that endometriosis can present itself as complete intestinal obstruction and should be considered when approaching women of childbearing age with acute abdominal pain.
